# Exploring pathways to digital transformation: fsQCA analysis based on the AMO framework

**DOI:** 10.1371/journal.pone.0315249

**Published:** 2024-12-27

**Authors:** Jun Liu, Ziwei Wang, Changjin Li, Ruofan Xu

**Affiliations:** School of Business Administration, Shandong University of Finance and Economics, Jinan, Shandong, China; Sichuan University, CHINA

## Abstract

In recent years, China has significantly increased its global competitiveness in digital technologies, emphasizing the importance of the digital economy during the high-quality development stage. The question of how firms in traditional industries can achieve digital transformation, which is critical for participating in the digital economy, is still understudied. Using the ability-motivation-opportunity (AMO) framework, this research developed a model and identified six factors’ ability, motivation, and opportunity dimensions. It used fuzzy-set qualitative comparative analysis (fsQCA) to investigate their synergistic effect on digital transformation. With manufacturing firms in China as examples, the findings were as follows. First, no single factor could provide the necessary conditions for digital transformation, implying that the synergistic effect of multiple conditions must be considered. Second, four configurations with three paths for high digital transformation, namely motivation-opportunity-oriented, total factor-oriented, and motivation-oriented, showed different approaches to digital transformation under different conditions. These findings shed light on the complex causal relationships among antecedents of digital transformation and provide theoretical and practical recommendations for businesses looking to implement the digital process.

## 1. Introduction

A report of the 20th National Congress of the Communist Party of China (CPC) proposed the task of “accelerating the development of digital economy, promoting the deep integration of digital economy and real economy, and building a digital industrial cluster with international competitiveness.” The rise and prosperity of the digital economy have created “new fields, new tracks, new drivers, and new advantages” for economic and social development, with China playing a leading role in both. With the rapid advancement of the Internet, big data, cloud computing, and artificial intelligence, digital technology and digital economy are becoming fully integrated into all aspects of economic, political, cultural, social, and ecological civilization construction, resulting in new development philosophy, forms, and modes [1–4].

Firms are important micro-foundations for developing the digital economy, so their digital transformation directly impacts speed and quality. Digital transformation is a process that seeks to improve an entity by causing significant changes to its properties through the use of information, computing, communication, and connectivity technologies [5], and it is deeply embedded in the operation and management of businesses. Digital transformation improves operational management decisions and efficiency [6]. An innovative mobile app recommendation strategy that considers users’ overall and current interests can effectively understand customer needs and provide accurate recommendations [7]. More importantly, digital transformation has the potential to redefine the competitive paradigm and revolutionize firms’ operational modes [6]. Due to the re-programmability of digital technology and data homogenization, firm boundaries have shifted. Innovation processes are less constrained, and the innovation agency becomes less predefined [8]. However, despite the potential benefits, many businesses have yet to achieve the expected results from digital transformation. 90% of them face challenges with timing, digital technology attributes, and a lack of opinion leaders [9].

As a result, a variety of factors affect digital transformation. For example, digital transformation requires digital resources, organizational structure, and digital growth strategies [10]. According to Porfírio et al. (2021) [11], effective management, including democratic leadership, consistent actions, and efficient strategic processes, promotes digital transformation. Governments promote digital transformation by developing digital platforms and offering digital training [12]. While research into the antecedent factors of digital transformation has yielded fruitful results, two significant deficiencies remain. First, previous research has emphasized that a single factor is required for high-level digital transformation, resulting in contradictory findings about the same outcome. For example, Wang et al. (2023) [13] argue that the positive aspiration performance gap (APG) has the advantage of meeting the financial requirements of digital transformation. In contrast, Zhu et al. (2023) [14] argue a U-shaped relationship between the positive APG and innovation input. The inconsistent findings encourage gaining a more comprehensive understanding of digital transformation. Second, traditional analytical techniques concentrate on a single variable’s marginal net effect while controlling other variables, ignoring digital transformation’s asymmetry and causal complexity. This limitation prevents us from determining the synergistic effect of multiple factors resulting in the same outcome.

The primary purpose of this research is to identify the influencing factors and explain their synergistic effect and interconnected relationship in driving digital transformation. It employed the ability-motivation-opportunity (AMO) framework to identify the influencing factors, which include the three fundamental concepts of ability, motivation, and opportunity. Ability refers to the skills, knowledge, and capabilities needed to complete a task. Motivation is the desire or impetus to achieve something. Finally, opportunity refers to the contextual or situational constraints that influence performance. The AMO framework is widely used to explain employee innovative performance at the individual level [15, 16], knowledge transfer [17], and organizational internationalization [18]. Digital transformation, like other strategic activities, is influenced by abilities, motivations, and opportunities [19].

The fuzzy-set qualitative comparative analysis (fsQCA) method investigates the synergistic effect of various factors in digital transformation. While regression analysis can only handle the marginal net effect of the explanatory variable when other variables remain constant, the fsQCA method can distinguish multiple conjunctural causations of antecedent conditions. It is especially well-suited to dealing with synergistic causal relationships between multiple variables [20, 21]. Furthermore, it has the advantage of distinguishing between core and peripheral conditions, which helps determine the effect of individual factors in various configurations. Therefore, it can reveal the relationship and synergistic effect between the ability, motivation, and opportunity factors of digital transformation and the configurations that determine digital transformation success or failure.

This research’s main findings were as follows. First, achieving digital transformation does not require a single antecedent condition. This research investigated the synergistic effects and interconnected relationships between different conditions. Second, this research identified four configurations with three paths for high-level digital transformation: motivation-opportunity-oriented, total factor-oriented, and motivation-oriented. Notably, the absence of financial constraints is a fundamental or peripheral condition in all configurations. These configurations, driven by different factors, provide viable options for businesses to achieve digital transformation in various situations.

This research’s sections are organized as follows. It reviews the literature on digital transformation and introduces the research model in Section 2. Section 3 proposes research hypothesis. Section 4 describes the research and data methodology, while Section 5 presents the results. Section 6 and Section 7 provide the discussion and the conclusion, respectively.

## 2. Related literature and research model

### 2.1 Literature review on digital transformation

Because of its interdisciplinary nature, the concept of digital transformation has piqued scholars’ interest. From a technological perspective, Cozzolino et al. (2018) [22] examined the disruptive impact of digital systems and technologies on incumbent firms. According to Li et al. (2017) [23], the strategic management perspective emphasizes that digital transformation is about more than just technology; it also includes fundamental changes in business processes, operational routines, and organizational capabilities. Hinings et al. (2018) [24] defined the institutional perspective of digital transformation as the combined effects of several digital innovations that produce new actors, structures, and other factors that either change, threaten, replace, or complement established rules. From a process perspective, Vial (2019) [5] defined digital transformation as a process that aims to improve an entity by causing significant changes in its properties through information, computing, communication, and connectivity technologies. This viewpoint distinguishes three primary digital transformation processes: digitization, digitalization, and digital transformation [10].

Digital transformation antecedents can be categorized into three levels. At the level of top managers and leadership, democratic leadership, managers’ coherence, and strategic planning efficacy trigger digital transformation [11]. Chief digital officers (CDOs) serve as entrepreneurs, digital evangelists, and digital process coordinators. The specific role of CDOs may differ depending on the firm’s maturity and employees’ digital mindset [25]. To address cognitive biases and perceptual blindness among senior executives in business-IT alignment, the chief information officer (CIO) has emerged as a strategic partner to the CEO [26]. Additionally, top managers who thoroughly understand digitalization, establish the formal context for digitalization, and lead change are better prepared to deal with digital transformation [27].

At the firm level, organizational resources and capabilities are critical. Dynamic capability not only directly contributes to process, product, and service digitalization, as well as business model digitalization [28], but it is also a strategic imperative for incumbent firms to survive in the digital age [29]. Furthermore, digital assets, digital agility, digital networking capabilities, and big data analytic capabilities are critical for achieving digital transformation [10]. Based on dynamic capability theory, integrative capability enables firms to keep up with changes in their value network during digital transformation [5].

External and environmental factors include institutional factors as well as market conditions. For instance, if firms want to realize the potential value of data and promote digitalization fully, the government should strengthen data security oversight and create a perfect commercial data transaction market system [30]. Government subsidies and tax incentives have a positive relationship with digital transformation because they relieve cost pressure during the early stages of digital transformation [31]. Moreover, enhancing the urban business environment may increase the likelihood and scope of digital transformation [32].

### 2.2 Research model

While the AMO framework is widely used in high-performance work contexts to explain individual creativity [33], it has also been extensively used in research on digital activities. According to the AMO theory, Li and Long (2023) [15] found that self-motivation with multiple supports, organization-driven with digital orientation, and digital-driven with ambidextrous complementary are the configurations that drive high employee digital innovation behavior. Yang et al. (2023) [34] argued that digital literacy (A), social relationships (O), and entrepreneurial passion (M) may be the process mechanisms that explain how digital penetration influences rural residents’ entrepreneurial behavior in tourism. According to Ojo et al. (2018) [35], digital skill is the most significant predictor of Internet usage, followed by opportunity and extrinsic motivation. In the digitalization of the tourism industry, AMO theory helps to understand the interaction conditions with cashless payment technology in tourist destinations [36]. From the literature findings and practical experience, this research created an integrated framework of ability, motivation, and opportunity dimensions and six secondary conditions to elucidate the factors influencing digital transformation. The three dimensions are provided below.

#### 2.2.1 Ability

The ability dimension includes two secondary conditions: human capital structure and innovation capability. According to human resource theory, human capital is the value of workers’ knowledge, technical skill, working ability, and physical health status combined. Technological advancements in digital transformation reduce the demand for low-tech and repetitive work, increasing the demand for highly skilled professionals with data analytics and digital networking skills [10]. Human resources with digital literacy are more adaptable in a dynamically changing work environment [37]. Furthermore, employees with high human capital levels can inhibit management’s myopia and actively promote digital transformation [38]. Employees with more educational experience can fully utilize the benefits of collective rationality and group wisdom in business collaboration to assist management in optimizing decision-making, thereby reducing the degree of irrationality in decision-making and preventing managers from abandoning investment in digital transformation due to risk aversion.

Innovation capability is a firm’s ability to develop new products and markets by aligning strategic, innovative orientation with innovative behaviors and processes, emphasizing the link between a firm’s resources and capabilities and the product market [39]. On the one hand, innovation capability provides the necessary software and hardware foundation for digital transformation, overcomes knowledge and technical barriers during the digital transformation process [40]. Moreover, it enables firms to absorb internal and external technology and strategy to incorperate the digital capability into objects that previously had a purely physical materiality [41], helping businesses make substantial transformations. On the other hand, firms with strong innovation capabilities will be more sensitive to digital information in both internal and external environments and more willing to experiment with high-risk, highly uncertain behaviors. Thus, they are more inclusive and willing to embrace digital transformation [42].

#### 2.2.2 Motivation

The motivation dimension includes two secondary conditions: top management team (TMT) characteristics and financial constraints. According to the upper echelons theory, individual experiences shape TMT cognition and knowledge, influencing strategic decision-making and firm performance [43]. This means top managers’ demographic characteristics significantly impact the firm’s strategic decisions. Top executives with overseas working or studying experience have a robust ability to understand and absorb advanced knowledge and skills [44], which helps them cope with the intense pressures of digital transformation [45]. Furthermore, given China’s scarcity of returnee managers, employees, the government, and investors expect returnee managers to be capable of leading the firm forward. This premise encourages returnees to focus on exploratory activities, demonstrating their abilities and advancing digital transformation.

Digital transformation requires ongoing investment in digital equipment and significant changes to business models and value chains, making it more than a technological upgrade. The firm faces significant financial constraints due to the long-term and uncertain transformation process and increased support facility requirements [46]. Firms in a relatively favorable financing environment tend to have a more optimistic expectation on their cash flow, which reduces their reliance on leveraged lending for investment and financing. It promotes the optimization of their financing structure and internal resource allocation, as well as a positive impact on firms’ digital transformation [47]. Moreover, as firms’ resource frontiers improve, their risk sensitivity decreases [48], indicating that top managers are motivated to shift their focus from maintaining the status quo to increasing their awareness of exploratory digital activities.

#### 2.2.3 Opportunity

The opportunity dimension comprises two secondary conditions: regional digital economy development and government subsidies. From an institutional standpoint, the digital economy introduces new actors, structures, practices, and values that alter, threaten, replace, or complement existing game rules in the fields [24], which would put normative and mimetic isomorphism pressure on firms. Firms that fail to adapt to changing operational norms risk falling behind and possibly losing their position on the development path. Moreover, imitating the pillar firms of digital transformation can be a cost-effective and time-saving strategy for gaining an advantage in the industry. Therefore, firms have incentives to initiate digital transformation in the regions with high level of digital economy.

As the net cash flow for firms, government subsidies can significantly alleviate the infrastructure and sunk cost pressure associated with a firm’s digital transformation investments in digital equipment upgrades and business model transformation [31]. More importantly, government subsidies serve as a signaling mechanism by providing innovation subsidies to specific firms. In the investigation and selection of funded firms, the government serves as an information intermediary, releasing signals to external investors and allowing them to differentiate the quality of firms at a lower cost. It helps reduce market information asymmetry [49, 50]. Therefore, firms can attract more resources to invest in digital technology research and development. Moreover, since the government monitors the use and purpose of the subsidies, funded firms consciously invest the grants in digital-related activities, creating a favorable environment for digital transformation [51].

**Fig 1:**
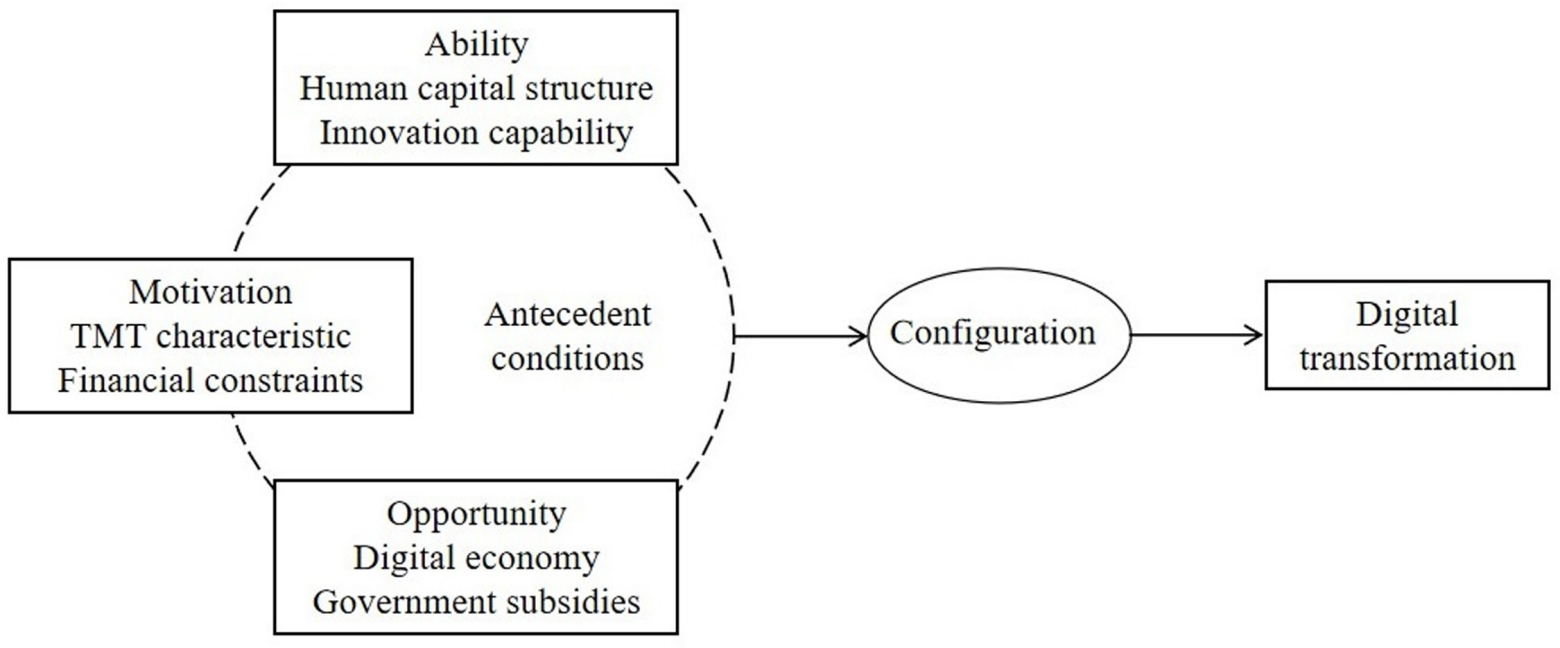
Research model.

In summary, the research framework is built around three dimensions (ability, motivation, and opportunity), as well as six conditions. These factors are highly interconnected rather than independent, resulting in synergistic effects on digital transformation. From a holistic standpoint, this research created a model as depicted in Fig 1.

## 3. Research hypothesis

Principal-agent conflict and information asymmetry problems impede digital transformation for the following reasons: First, digital transformation encompasses not only technology upgrades and equipment updates but also the transformation of the business model. Because high specialization and a long payback period make it difficult to estimate the short-term benefits of digital transformation, executives with a risk-averse orientation will lower their expectations for the benefits. Therefore, shareholders with a long-term perspective will experience principal-agency conflicts with executives, increasing the cost of digital transformation. Second, digital transformation’s high specialization and long-term nature raise firms’ review and regulatory costs for external investors [52]. Moreover, firms may be hesitant to disclose internal information due to concerns about protecting patents and technical secrets, resulting in an information asymmetry between external investors and firms. In this scenario, investors raise the capital premium to mitigate risks, making it difficult for businesses to obtain adequate external financing for digital transformation.

The apparent effect of reducing financing constraints is to meet the demands of technology upgrades and equipment updates in digital transformation. Furthermore, alleviating financing constraints improves the financing environment, giving firms more optimistic cash flow forecasts and lowering financial risks. Improving enterprises’ financial structures boosts external investor confidence, thereby mitigating the information asymmetry problem. As resource constraints are alleviated, executives’ limited attention will shift from reducing financial risks to digital transformation. Focusing on digital transformation will reduce executives’ risk aversion and myopia, improve the consistency of goals and interests between shareholders and executives, and thus promote digital transformation.

Based on the preceding analysis, the hypothesis is as follows: *Digital transformation requires low financing constraints to alleviate principal-agent conflict and information asymmetry*.

## 4. Methodology

### 4.1 Research method

The QCA method, which combines qualitative and quantitative techniques, has received increased attention. Based on Boolean logic operation, it is commonly used to analyze the connection of complex elements and the combination path of antecedents. Currently, this method is widely used in management. When compared to traditional statistical methods, it is not only appropriate for small-sized samples but also for medium- and large-sized samples, which not only overcomes the poor external generalization of qualitative analysis but also compensates for the shortcomings of quantitative analysis that are insufficient for individual uniqueness [53].

This research used the fsQCA method for the following reasons. First, fsQCA can investigate the complex causal mechanisms underlying various factors influencing firm digital transformation. While multiple regression and other quantitative methods focus on the marginal net effect of explanatory variables with other variables held constant, fsQCA focuses on multiple conjunctural causations composed of various combinations of conditions on results based on configuration. Investigating the relationship and synergy between six interdependent internal and external factors in a firm’s digital transformation is appropriate. Second, unlike mvQCA and csQCA, fsQCA considers the degree and specific membership and the truth table’s advantages in dealing with qualitative data, limited diversity, and simplified configuration [53]. It is consistent with this research’s diverse characteristics of condition data types (both dummy and continuous). Third, combining the AMO framework and the fsQCA approach has effectively clarified the internal mechanisms that drive strategic activities [18, 19]. Thus, the fsQCA approach used in this research investigates how ability, motivation, and opportunity conditions influence digital transformation. The fundamental analysis of fsQCA includes the following steps: calibration that assigns fuzzy-set membership scores to sample cases, followed by necessity analysis and sufficient analysis of the configurations.

### 4.2 Sample cases selection and data source

The QCA method uses theoretical sampling rather than random sampling to avoid sample selection bias [54]. Since 2015, The State Council of China has issued “Made in China 2025” and “on Deepening the Integrated Development of manufacturing and the Internet,” implementing digital transformation in the manufacturing industry. In recent years, China’s Ministries of Industry and Information Technology and Finance have issued the Intelligent Manufacturing Development Plan (2016–2020) and the Industrial Internet Development Action Plan (2018–2020), clarifying the specific goals and critical tasks of digital manufacturing transformation. The year 2019 marks the end of the policy, indicating a transition from Industry 2.0 to Industry 3.0. In comparison to previous years, the manufacturing industry has made significant progress. Therefore, the manufacturing industry in 2019 was chosen as the subject of this research.

Specifically, given the significant differences in the development paths of digital-born and traditional firms in digital transformation, using all types of firms as research samples would be inappropriate. Therefore, this research concentrated on traditional industrial firms while excluding digital-born ones such as computer, communication, and electronic equipment manufacturers. It gathered all the data from the China Stock Market and Accounting Research (CSMAR) Database. Furthermore, it excluded companies with distinct treatment labels indicating abnormal business conditions or incomplete financial information.

### 4.3 Measures

#### 4.3.1 Outcome variable

Digital transformation (DT). The words and expressions in the annual report reflect the firm’s development orientation, strategic characteristics, and outlook. This study used textual analysis to determine the frequency of digital transformation-related keywords in the annual reports of sample firms. Keywords for digital transformation include artificial intelligence, blockchain, cloud computing, big data, and digital transformation applications [55]. Taking the keyword frequency count’s logarithm helps mitigate the potential issue with the right-skewed distribution.

#### 4.3.2 Antecedent variables

Human capital structure (HC). Human capital is critical for long-term economic growth and development, and higher education is required to produce highly competent experts. The percentage of undergraduate or higher education employees is a proxy for a firm’s human capital structure.

Innovation capability (IC). This research assessed innovation capability by adding the natural logarithm of one to the total number of patents a firm owns, which includes invention, design, and utility model patents.

TMT characteristic (TMT). According to the upper echelons theory, TMT’s cognitive and psychological characteristics, as shaped by individual experiences and other demographic characteristics, impact strategic decision-making and firm performance [43]. Overseas experience is defined as working or studying abroad, and it is coded as one if top managers have such experience and zero otherwise.

Financial constraints (SA). The SA index measures the degree of financial constraint. A higher absolute value of this index indicates that firms face more severe financial constraints.

Regional digital economy development (DE). Following Meng et al. (2022) [56], this research developed an evaluation indicator system and a composite digital economy index to assess the digital economy’s development level. The evaluation indicator system includes five indicators: (1) the number of broadband Internet access users per hundred people; (2) the proportion of employees in the computer service and software industry; (3) total telecom business per capita; (4) the number of mobile phone users per hundred people; and (5) the China Digital Financial Inclusion Index, which the Peking University Digital Finance Research Center and Ant Financial Group calculate.

Government subsidies (GS). Government subsidies are measured by adding the natural logarithm of one to the subsidies received by the firm from the government, particularly those related to digital construction.

## 5. Results

### 5.1 Regression analysis

Before applying the QCA method, this research used regression analysis to identify the key antecedent conditions that significantly impact digital transformation. Regression analysis and the QCA method can provide a more comprehensive foundation for model interpretation [57]. As shown in Table 1, the descriptive and correlation analyses of antecedent conditions revealed no significant multicollinearity among variables (mean VIF = 1.12). Digital transformation strongly correlated with innovation capability, financial constraints, regional digital economy development, and government subsidies. Presumably, these four antecedent conditions may be critical. Innovation capability, regional digital economy development, and government subsidies correlated significantly positively with digital transformation, while financial constraints correlated negatively.

**Table 1:** Descriptive statistics and correlation analysis.

conditions	DT	HC	IC	SA	TMT	DE	GS	MEAN	SD
DT	1							1.262	1.150
HC	-0.0230	1						0.223	0.168
IC	0.207***	0.171***	1					4.853	1.243
SA	-0.086*	0.0130	-0.0540	1				3.912	0.225
TMT	0.0130	0.0660	0.082*	-0.0740	1			0.220	0.415
DE	0.098**	0.0670	0.0390	0.084*	-0.00600	1		0.382	0.294
GS	0.141***	0.141***	0.476***	-0.0120	0.0710	-0.0360	1	16.30	1.698

*, **, and *** indicate significance at the 0.10, 0.05, and 0.01 levels.

Table 2 shows how individual antecedents in the regression analysis affect the results. The regression coefficients for innovation capacity, regional digital economy development level, and government subsidy were all significantly positive. In contrast, the coefficient for financial constraints was negative, implying that financial constraints negatively impacted digital transformation. However, traditional quantitative analysis could not determine the impact of various combinations of conditions on the results. Furthermore, human capital and TMT characteristics were insignificant in the regressions, implying that the impact of these two conditions could not be identified in an independent analysis setting. It necessitated additional analysis using the QCA method.

**Table 2:** Regression analysis of antecedent conditions.

	Coefficient	t statistics	P-value
HC	-0.156	-0.488	0.626
IC	0.191	4.517	0.000
SA	-0.441	-1.851	0.065
TMT	0.035	0.273	0.785
DE	0.383	2.101	0.036
GS	0.095	3.042	0.002

### 5.2 Data calibration and necessary conditions analysis

Data calibration determines which cases belong to which sets. This research needed to convert the raw data into set membership scores ranging from 0 to 1. It employed direct calibration methods to obtain the fuzzy membership scores [58], specifically that the 85%, 50%, and 15% quantiles of the raw data would be the full membership, the cross-over point, and the full nonmembership anchor points, respectively. Table 3 presents the calibration anchor points for antecedent conditions.

**Table 3:** Calibration anchor points.

Conditions and outcome		Full membership	Cross over point	Full nonmembership
Ability	HC	0.34	0.18	0.09
	IC	6.17	4.92	3.74
Motivation	TMT	1.00	/	0.00
	SA	4.14	3.91	3.67
Opportunity	DE	0.77	0.28	0.10
	GS	18.00	16.00	15.00
Outcome variable	DT	2.56	1.10	0.00

Necessary conditions analysis determines whether a single condition is required for the outcome. If a condition’s consistency exceeds the benchmark of 0.9, it is necessary for the outcome [59]. The consistency of the presence and absence of any single condition with the high and non-high digital transformation results in Table 4 did not exceed the 0.9 benchmark. It entailed that the necessary conditions required to generate high and low digital transformations did not exist. To better understand digital transformation configurations, the synergistic effect and interconnectedness of ability, motivation, and opportunity conditions must be considered.

**Table 4:** Necessary conditions analysis.

Condition variables	DT		∼DT	
	Consistency	coverage	Consistency	coverage
HC	0.564	0.576	0.566	0.580
∼HC	0.589	0.574	0.587	0.575
IC	0.632	0.650	0.494	0.510
∼IC	0.523	0.508	0.661	0.643
TMT	0.226	0.514	0.214	0.486
∼TMT	0.773	0.495	0.786	0.505
SA	0.553	0.554	0.607	0.611
∼SA	0.612	0.608	0.556	0.555
DE	0.589	0.611	0.544	0.567
∼DE	0.582	0.560	0.627	0.605
GS	0.687	0.616	0.577	0.519
∼GS	0.464	0.522	0.573	0.647

### 5.3 Sufficient conditions and configurations of high digital transformation

The first step is to create a truth table to ensure the adoption of sufficient conditions with meaningful configurations [58]. Using the QCA algorithm, the truth table shows all possible configurations of conditions in 2k rows, where k represents the number of conditions. The parameters are then set as follows: Due to the large number of cases in the study, the case frequency cutoff was set to 4, excluding less significant configurations [58]. Furthermore, the raw consistency cutoff was set to 0.8 to ensure an adequate number of configurations for outcomes [58]. The proportional reduction in inconsistency (PRI) cutoff was set to 0.6 based on the distribution of the research samples to address concurrent subset relationships of configurations in the outcome [59]. This research then looked at complex, parsimonious, and intermediate solutions for high-level digital transformation. Conditions that appeared in intermediate and parsimonious solutions were considered core conditions, while those that appeared only in intermediate solutions were classified as periphery conditions [53]. Table 5 shows the conditional configurations for high digital transformation.

**Table 5:** Configurations of high digital transformation.

Condition variables	high digital transformation(with case frequency cutoff = 4, raw consistency cutoff = 0.8, PRI cutoff = 0.6)			
	H1	H2	H3a	H3b
HC	**⨂**		**⨂**	**⨂**
IC	**⨂**	●	⚫	⚫
TMT	⊗	**⨂**	●	●
SA	**⨂**	**⨂**	⊗	⊗
DE		●	⚫	⊗
GS	●	●	⊗	⚫
Consistency	0.804	0.792	0.861	0.843
Raw coverage	0.156	0.174	0.038	0.044
Unique coverage	0.084	0.101	0.022	0.029
Solution coverage	0.324			
Solution consistency	0.790			

Note: ● and **⨂** represent the presence and absence of core conditions, respectively; ⚫ and ⊗ represent the presence and absence of peripheral conditions, respectively; blanks represent that the condition is unimportant to the outcome.

Table 5 shows four configurations of three paths driving high digital transformation, numbered H1 through H3. The solution consistency of each configuration exceeded 0.75, indicating high reliability. The overall solution consistency was 0.790, exceeding the 0.75 benchmark and implying that these four configurations were sufficient conditions for high-level digital transformation. Moreover, the overall solution coverage of 0.324 indicated that these four configurations could represent 32.4% of high-level digital transformation cases.

Path 1: motivation-opportunity-oriented. Configuration H1 represents the motivation-opportunity-oriented type, as indicated by ∼HC*∼IC*∼TMT*∼SA*GS. This research identified government subsidies, the absence of financial constraints, human capital structure, and innovation capability as the core causal conditions. Despite their inability to initiate digitalization, firms can achieve high digital transformation with the assistance of a supportive government and a favorable financial environment. This configuration explained 8.4% of firms with high digital transformation, accounting for 15.6% of the sample firms.
Path 2: motivation-ability-opportunity-oriented (total factor-oriented). Configuration H2 represents the motivation-ability-opportunity-oriented type, as indicated by IC*∼TMT*∼SA*DE*GS. This research identified the presence of innovation capability, regional digital economy development, government subsidies, and the absence of financial constraints and returnee managers as the core causal conditions. This path suggests that firms with no financial constraints and strong innovation capabilities can achieve digital transformation in an environment marked by high digital economy development and government support, even without returnee top managers. This configuration explained 17.4% of sample firms and 10.1% of firms with high-level digital transformation can only be explained by this configuration.
Path 3: motivation-oriented. Configurations H3a and H3b represent the motivation-oriented type, as respectively indicated by ∼HC*IC*TMT*∼SA*DE*∼GS and ∼HC*IC*TMT*∼SA*∼DE*GS. These configurations are second-order equivalents because their core conditions are identical, yielding the highest consistency of the four configurations. With the presence of peripheral conditions of innovative capability and digital economy development or government subsidies, returnee top managers will be motivated to engage in the digital process. In particular, government subsidies and the development of the digital economy have a mutual substitution effect.

Regarding the analysis of overlapping paths and potential substitution relationships, this research examined the substitution relationship between overlapping paths and antecedent conditions in configurations 1, 2, and 3. Configurations 1 and 2 overlapped with the conditions ∼TMT, ∼SA, and GS. This premise implied a substitution relationship between ∼HC, ∼IC and IC, DE. Second, between configurations 2 and 3b (configurations 3a and 3b as second-order equivalents), IC, ∼SA, and GS were overlapping paths. Under this assumption, a substitution relationship existed between ∼TMT, DE and ∼HC, TMT, ∼DE. Third, between configurations 1 and 3b, ∼HC, ∼SA, and GS were overlapping paths. This premise suggested a substitution relationship between ∼IC, ∼TMT and IC, TMT, ∼DE.

### 5.4 Robustness test

This research used two approaches to test the results’ robustness based on set-theoretic principles. These methods included changing the case frequency cutoff from 4 to 5 and the raw consistency cutoff from 0.8 to 0.82. Theoretically, these changes would reduce the number of cases in the truth table, resulting in new configurations that are subsets of existing ones. The increase in case frequency and raw consistency cutoffs ensured that the high digital transformation configurations in Table 6 remained subsets of those in Table 5, implying that the configurations had not changed significantly. Thus, the results were robust [60].

**Table 6:** Robustness test.

Condition variables	high digital transformation(with case frequency cutoff = 5,consistency cutoff = 0.8, PRI cutoff = 0.6)			high digital transformation(with case frequency cutoff = 4,consistency threshold = 0.82, PRI cutoff = 0.6)		
	H1	H2	H3	H1	H2a	H2b
HC		**⨂**	**⨂**	**⨂**	**⨂**	**⨂**
IC	●	**⨂**	●		⚫	⚫
TMT	**⨂**	⊗	●	⊗	●	●
SA	**⨂**	**⨂**	⊗	**⨂**	⊗	⊗
DE	●	⊗	**⨂**	●	⚫	⊗
GS	●	●	⚫	●	⊗	⚫
Consistency	0.792	0.819	0.843	0.798	0.861	0.843
Raw coverage	0.174	0.128	0.044	0.151	0.038	0.044
Unique coverage	0.113	0.067	0.044	0.151	0.022	0.029
Solution coverage	0.286			0.218		
Solution consistency	0.794			0.815		

## 6. Discussion

Based on the AMO framework, this research’s primary goal was to identify the antecedent factors and their synergistic effects on digital transformation. Taking a holistic approach, even if firms do not meet all the conditions, they can still achieve digital transformation through the synergistic effects of ability, motivation, and opportunity conditions. This lens provides valuable insights for furthering the understanding of digital transformation in traditional manufacturing firms.

First, the absence of financial constraints as a core or peripheral condition in the four configurations of high digital transformation confirms that digital transformation necessitates significant physical and financial investment in equipment upgrades and platform development [46]. Moreover, removing financial constraints improves the financing environment, giving businesses a more positive outlook on cash flow. It boosts external investor confidence while also addressing information asymmetry. Because of the resolution of financial issues, executives’ limited attention shifts from reducing financial risks to digital transformation, which helps to alleviate risk aversion and myopia among executives. Thus, aligning goals and interests between shareholders and executives will improve, promoting digital transformation. Furthermore, this research demonstrated that a lack of financial constraints does not preclude digital transformation, implying a complex causal relationship rather than a linear relationship between financial constraints and digital transformation.

Second, aside from financial constraints, Path 1 (motivation-opportunity-oriented) emphasizes the importance of government subsidies in digital transformation. Government support can not only directly assist firms in meeting the financial requirements of digital input, but it can also serve as a signal. Obtaining government subsidies is an essential signal that the funded firms have development potential. It reduces the information asymmetry between firms and investors caused by the uncertainty of digital activities, allowing firms to obtain additional financing support. Furthermore, a favorable innovation environment would attract more highly skilled professionals to agglomerates [61], improving the firm’s human capital structure. That means government subsidies compensate for the absence of organizational capabilities in the digital transformation process.

Third, Path 2 (total factor-oriented) has the highest raw coverage of any configuration, emphasizing the significance of the synergistic effects of ability, motivation, and opportunity factors. Government subsidies to obtain policy resources and gain competitive advantages can mitigate the disadvantages of insufficient resources or poor utilization ability. The institutional perspective holds that the institutional environment in which firms operate impacts organizational behavior, implying that the legitimacy of the institutional environment is a critical factor influencing firm behavior. The growth of regional digital economies primarily influences digital transformation via normative and mimetic isomorphism. On the one hand, the digital economy’s growth has gradually altered market operating procedures and rules. If firms do not actively adapt to the legitimacy pressures posed by the new system operation paradigm, they will struggle to conduct production and trading in the region and risk being expelled from the ecosystem [62, 63]. On the other hand, a change in business model alters the regional market’s competition standard, and pursuing a competitive position encourages imitation behavior among firms. Firms are likelier to imitate the industry’s pillar firms during the digital transformation to gain competitiveness quickly.

The resource-based view holds that firms’ competitive advantage in growth stems from their resource endowment. Firms with strong internal resources can form a resource position barrier and gain unique competitive advantages [64]. Innovation capability is a dynamic capability of firms that is valuable, scarce, and difficult to replicate. It can help businesses gain a sustainable competitive advantage.

Finally, Path 3 (motivation-oriented) focuses on the role of top managers in digital transformation. Top managers such as CDOs play multiple roles in digital transformation, including entrepreneurs, digital evangelists, and coordinators [25]. Collaboration between the CIO and CEO is critical for successful business-IT alignment [26]. Returnee top managers with open minds and a willingness to take risks can assist employees in becoming more engaged in digital transformation and less committed to the status quo by providing innovation skills and a supportive external environment. It can help compensate for the shortage of advanced human capital. Furthermore, government subsidies and digital economy development have a mutual substitution effect. The underlying reasons could be as follows. When the government distributes subsidies to regions with underdeveloped digital facilities and a small digital economy, increasing resource input promotes scale efficiency and digital transformation, a phenomenon known as “timely assistance”. On the contrary, in regions with a high level of the digital economy, excessive increases in resource input may have restricted marginal net effect in promoting digital transformation due to the shock to a stable input structure, much like icing on the cake.

## 7. Conclusions

Using the AMO framework, this research identified six conditions based on the dimensions of ability, motivation, and opportunity to build an integrated digital transformation model. Following that, the fsQCA method investigated how the antecedent conditions are related and how they interact to produce high levels of digital transformation. The key findings are as follows. First, no single condition is required to achieve digital transformation. Second, three paths contribute to high-level digital transformation: motivation-opportunity-oriented, total factor-oriented, and motivation-oriented. Specifically, the absence of financial constraints is the core or peripheral condition in all configurations. Path 1 emphasizes the role of government subsidies as a catalyst for high-level digital transformation. Path 2 emphasizes the synergistic effect of all three dimensions in transforming the digital business model and value creation processes. Finally, Path 3 focuses on the role of top managers. In particular, government subsidies have a mutual substitution effect on the development of the digital economy.

### 7.1 Theoretical contributions

This research has multiple contributions. First, it created an integrated model using prior research on the antecedents of digital transformation and the AMO framework. The model identified digital transformation’s ability, motivation, and opportunity factors. Unlike the technology-organization-environment (TOE) framework, which focuses on analyzing the antecedents of digital activities [65], this research broadened the perspective on the antecedents of digital transformation by identifying them using the AMO framework. Furthermore, it broadened the AMO framework’s application scope in digital transformation research and validated its effectiveness beyond traditional scenarios. Moreover, by applying the AMO framework to the unique Chinese management context, this research validated the AMO’s applicability and malleability as a meta-theory.

Second, previous research examined the significant impact of individual antecedent conditions on digital transformation, emphasizing the importance of each condition. In contrast, this study reveals that the conditions required for digital transformation do not exist. Even if the absence of financial constraints is the core or peripheral condition, high-level digital transformation can only be accomplished through the combined efforts of several factors. This comprehensive research approach considers multiple conditions simultaneously, which aids in identifying the underlying causes of inconsistent conclusions resulting from the same single condition.

### 7.2 Practical implications

The findings of the research have managerial implications as follows:

For governments and official authorities, there are two main actions they should take. First, they are supposed to formulate attractive policies such as tax breaks, subsidized grants and loans, and digital training programs. These measures can create a favorable environment for firms to tackle with challenges related to funding, digital capability, and human resources in the process of digital transformation. Second, local governments should actively participate in the digital economy by constructing digital platforms, infrastructures, and fostering the digital ecosystem. These efforts can assist traditional firms in achieving successful digital transformation.

For firms, initiating and managing digital transformation can be challenging due to high risk, unpredictable ambiguities, and strong resistance to change. It is crucial to alleviate financial constraints in the realization of digital transformation. Firms should actively seek additional resources, such as financial support from governments and financial institutions, to overcome resource constraints in digital transformation directly. Moreover, financial constraints motivate firms to operate under budget constraints, prompting them to improve the effectiveness and efficiency in reallocation and exploitation of resources.

For top managers, there are two key aspects to consider. First, they should adopt a holistic perspective and recognize the multiple driving forces of digital transformation. Having a comprehensive understanding of the pace and steps involved in digital transformation is essential for its successful implementation. Second, top managers should continuously enhance their capabilities and broaden their horizons by absorbing cutting-edge knowledge and management skills. This will enable them to effectively interpret digital information and seize digital opportunities.

### 7.3 Limitations and future research

There are three limitations that warrant further exploration. First, digital transformation is a multidimensional concept encompassing diverse antecedents and outcomes. It is essential to differentiate dimensions such as depth and breadth of digital transformation. Future research could integrate other methods, such as questionnaire analysis, to comprehensively evaluate digital transformation. Second, as there are notable differences between QCA and traditional analytical methods, it is highly recommended to integrate the set-theoretic methods with other analytical methods. The integration would enhance the explanatory power of the research findings. Third, although this research develops an integrative model based on the AMO framework to examine the antecedents and their relationships, numerous factors remain under-explored. Future research should aim to integrate additional theoretical frameworks to explore the pathways to digital transformation.

## Supporting information

S1 Dataset The data set used in this paper for discussion and analysis. (ZIP)
